# Political Prioritisation for Performance-Based Financing at the County Level in Kenya: 2015 to 2018

**DOI:** 10.34172/ijhpm.2023.6909

**Published:** 2023-02-14

**Authors:** Dennis Waithaka, Lucy Gilson, Edwine Barasa, Benjamin Tsofa, Marsha Orgill

**Affiliations:** ^1^Health Economics Research Unit, KEMRI-Wellcome Trust Research Programme, Nairobi, Kenya.; ^2^Health Policy and Systems Division, School of Public Health and Family Medicine, University of Cape Town, Cape Town, South Africa.; ^3^Department of Global Health and Development, Faculty of Public Health and Policy, London School of Hygiene and Tropical Medicine, London, UK.; ^4^Centre for Tropical Medicine and Global Health, Nuffield Department of Medicine, University of Oxford, Oxford, UK.; ^5^Health Systems Research Group, KEMRI-Wellcome Trust Research Programme, Kilifi, Kenya.

**Keywords:** Performance-Based Financing, Politics, Political Prioritization, Power, Sub-national, Kenya

## Abstract

**Background:** Performance based financing was introduced to Kilifi county in Kenya in 2015. This study investigates how and why political and bureaucratic actors at the local level in Kilifi county influenced the extent to which PBF was politically prioritised at the sub-national level.

**Methods:** The study employed a single-case study design. The Shiffman and Smith political priority setting framework with adaptations proposed by Walt and Gilson was applied. Data was collected through document review (n=19) and in-depth interviews (n=8). Framework analysis was used to analyse data and generate findings.

**Results:** In the period 2015-2018, the political prioritisation of PBF at the county level in Kilifi was influenced by contextual features including the devolution of power to sub-national actors and rigid public financial management structures. It was further influenced by interpretations of the idea of ‘pay-for-performance’, its framing as ‘additional funding’, as well as contestation between actors at the sub national level about key PBF design features. Ultimately PBF ceased at the end of 2018 after donor funding stopped.

**Conclusion:** Health reformers must be cognisant of the power and interests of national and sub national actors in all phases of the policy process, including both bureaucratic and political actors in health and non-health sectors. This is particularly important in devolved public governance contexts where reforms require sustained attention and budgetary commitment at the sub national level. There is also need for early involvement of critical actors to develop shared understandings of the ideas on which interventions are premised, as well as problems and solutions.

## Background

###  Performance-Based Financing Introduction in Low- and Middle-Income Countries

**Key Messages**

** Implications for policy makers**

- To influence and sustain the political prioritisation of reforms in devolved contexts, adequate, early involvement and leadership from sub-national bureaucratic and political actors is required, from both health and other sectors.
- The ways in which ideas underpinning reform resonate with actors influences its relevance for policy-makers. Early involvement of critical actors is important in developing a common understanding of the ideas underlying the reforms, problem(s) being addressed and potential alternative solutions.
- The political context, including factors like public financial management (PFM) laws and the ruling political party, is an important feature of reform, as it influences the acceptability and political prioritisation of reforms.

** Implications for the public**

Performance-based financing (PBF) aims at improving the quantity and quality of healthcare services provided to the population by providing cash rewards to health providers if they achieve agreed targets. The study investigated how and why sub-national actors influenced the political prioritisation of PBF. Our findings draw attention to the influence of the political environment, ideas and practices of power at different levels of governance, and their impact on the prioritisation and adoption of health sector reforms. The work can help deepen an understanding of the complexities of policy processes that ultimately impact access to the type of services the public receives.

Performance-based financing (PBF) is “a form of service provider payment where financial incentives are directed to healthcare providers (not beneficiaries) when they achieve pre-determined process or output indicators, adjusted by some measure of quality.”^1^ It represents a shift away from the traditional payment for inputs (such as staff salaries and drugs), which has been argued to be inefficient due to, for example, resource leakage, lack of accountability and staff absenteeism.^2-7^

 Some evidence indicate that PBF promotes rapid improvement, at least in the short-term, for simple and well-articulated behavioural goals,^8,9^ as well as strengthens governance structures and strategic purchasing.^4,10,11^ However, PBF has been contested both on ideological and practical grounds.^7^ Some have criticized PBF for attempting to quantify (and price) complex health system processes into single indicators. Others have claimed that it leads to inequitable access to healthcare services as health workers are more likely to focus on the incentivised indicators and services whilst reducing quantity and/or quality of services delivered for the non-incentivised indicators.^7^ Critics have also argued that the rapid and widespread introduction of PBF in low- and middle-income countries is a result of strong advocacy from international consultants and organisations, ignoring the contradictory evidence on its effectiveness and efficiency in these settings.^12,13^ In practice, the widespread introduction of PBF in low- and middle-income countries, including sub-Saharan Africa, has rarely been accompanied by its successful evolution into national public policies.^6^ While there is growing evidence about the technical challenges of implementing PBF schemes in sub-Saharan Africa (eg, Antony et al,^14^ Ridde et al,^15^ Zittiet al^16^), there is a limited body of literature about the political economy and politics of PBF policy processes in these settings. Available studies have focused at global/continental,^17^ regional^18^ and/or national levels.^17,19-21^ This study seeks to contribute to this limited literature by examining the experience of PBF political prioritisation at the sub-national, or county, level in Kenya.^22^ The term political prioritisation refers to the process through which an issue gains sustained attention by political leaders who then allocate resources that match the severity of the issue.^23^

###  The Introduction of PBF in Kenya: The Political and Policy Environment

 The Kenya Health Sector Support Project (KHSSP) was a health sector financing project agreed between the World Bank and the Government of Kenya in 2010.^24^ One of its aims was to improve the delivery and utilisation of quality essential health services to women and children, especially among poor and drought-affected populations (that is, arid and semi-arid land [ASAL] regions). This would be achieved by providing funding to support the implementation of the Health Sector Service Fund (HSSF) and PBF.^24^ The HSSF ensured that cash funding reached primary healthcare (PHC) facilities by transferring funds for day-to-day expenses directly from the national treasury to PHC facility accounts.^25^ This mechanism of direct cash transfer sought to address historical challenges faced by PHC facilities – of very limited and delayed funding due to “bureaucratic reasons, leakages in financial flows or diversion to other priorities.”^26^ PBF was intended to use the HSSF structures to transfer funds directly from the national treasury to the PHC facility accounts.^24,27^ The underlying premise was that the provision of financial incentives targeted at maternal and child health (MCH) services (see Supplementary file 1 for the specific indicators) in PHC facilities, through PBF, would motivate health workers to improve performance and thereby “accelerate” the achievement of the Millennium Development Goals 4 and 5.^27^

 In the year that KHSSP implementation began (2010), a new constitution was also adopted in Kenya. It provided for the creation of a devolved system of governance comprising a national government and 47 semi-autonomous county governments.^22^ Table 1 shows the county level government structures and responsibilities post-devolution. This new governance system granted enhanced decision-making powers to county level actors, influencing how political and bureaucratic actors in Kilifi county shaped the prioritisation of PBF in their county.

**Table 1:** County Level Government Structures and Responsibilities Post-devolution

**County Government Structures**	**Actors Within the County Structures**	**Key Responsibilities**
County legislature	Elected MCAs.	Make any county legislation necessary for the effective functioning of the county government. Oversight over the county executive. Receive and approve plans, budgets and policies, for the management and utilisation of county resources and institutions.
County executive	Elected county governor and deputy governor. CEC officials for each county department (are individuals with the knowledge and experience relevant to manage their department, and are appointed by the governor and approved by the county assembly). Notably, working under the CEC official is a Chief Officer, also appointed by the governor and is responsible for the day-to-day functioning of the department.	Implement national and county legislation. Manage and coordinate the functions of the county administration and departments. Provide the county assembly with full and regular reports on matters related to the county. Prepare proposed legislation for consideration by the county assembly.

Abbreviations: MCAs, members of the county assembly; CEC, County executive committee. Source: Government of Kenya.^22^

 The devolved system of governance was adopted after the March 2013 national and county government elections, and the initial intention was to transfer functions progressively from the national government to counties with guidance from a transition authority.^25^ Post-devolution, the national Ministry of Health (MoH) is assigned the roles of policy and standards formulation, management of national referral services and pre-service training for health workers. The county governments are, meanwhile, assigned all health service delivery functions including the management of human resources for all facilities within a county (this excludes national referral hospitals).^22^ Within this system, although the national MoH held responsibility for the formulation of the PBF policy, the county governments had the legislative power to determine whether any legislative actions and/or resource allocation to health facilities under the banner of PBF, would occur at the county level.^22^ Ultimately, then, county actors determined the extent of prioritisation accorded to the programme.

 However, soon after the March 2013 elections, the newly elected governors pushed for the immediate transfer of all county functions.^25^ In June 2013, the President reportedly “succumbed” to this political pressure and “directed” the immediate transfer of all devolved functions to the counties despite their lack of capacity and structures to undertake the functions at the time.^25^ This resulted in lack of clarity and contestation over the “specific” responsibilities of the national and county governments and their entities/departments (eg, for procurement of health commodities, management of intercounty transfers for health workers, in-service training and career progression).^25^ Most notable was the country-wide contestation between the MoH and county governments over their roles in the management and channelling of the HSSF funds.^26^ The contestation was linked to the introduction of a new public financial management law (PFM Act of 2012) that brought about changes in public financial management structures and processes. Pre-devolution, financial management had been decentralised to healthcare facilities and facilities were allowed to operate bank accounts, receiving funds directly into these accounts and having oversight over these funds.^28^ However, upon devolution, the new PFM Act^29^ recentralised financial management from health facilities to the level of the county treasury, with all funds managed in a centralized account at the county treasury – the county revenue fund.^30^

 In this study, we will focus on the evolution of key events from early 2015 when PBF was communicated to Kilifi county actors by the national MoH up until the short four month period of PBF implementation in Kilifi in 2018. The analysis concludes at the end of 2018 when donor funding for PBF ceased and the implementation of PBF ended in Kilifi county. Our research question is: how and why did sub-national political and bureaucratic policy actors influence the political prioritisation and adoption of PBF, a national policy, in Kilifi county?

## Methods

###  Conceptual Framework

 The overarching framing for the study was conceptualised by DW, MO, and LG. The study adopted the Shiffman and Smith^23^ political priority setting framework, including adaptations by Walt and Gilson^31^ (see Table 2). It has previously been used to examine why some global health issues are more (or less) successful in generating political priority at the global^23,32,33^ and national^34,35^ level. We apply this framework at the sub-national level in this study. Political prioritization is present when: (1) international and national political leaders publicly and privately express sustained concern for the issue; (2) the organisations and political systems they lead enact policies to address the problem; and (3) these organisations and political systems provide levels of resources to the problem that are commensurate with its severity.^23^

**Table 2:** The Shiffman and Smith^23^ Political Priority Setting Framework, Including Adaptations (Changes) Proposed by Walt and Gilson^31^

**Categories**	**Description**	**Factors Shaping Political Priority**
Actor power	The strength of the individuals and organisations concerned with the issue	1. Policy community cohesion 2. Leadership 3. Effective guiding *‘ organisations ’ (as proposed by*Walt and Gilson^31^ *to replace the term ‘institutions’)* 4. Civil society mobilisation
Ideas	The ways in which those individuals with the issue understand and portray it	5. Internal frame 6. External frame
Issue characteristics	Features of the problem	7. Credible indicators 8. Severity 9. Effective interventions 10. *Contestations or conflicts*
Political contexts	The environment in which actors operate	11. Policy windows 12. Global *and national* governance structures *(formal and informal institutions)* 13. *Historical dimension*
*Outcome*	*Assessment of whether the issue is being taken seriously by policy-makers*	*12. Presence of an authoritative decision or resources allocated to issue*

Note: Additions or changes made by Walt and Gilson^31^ highlighted in *italic*.

 Generating political priority is understood to be more likely when the actors who are concerned with the issue have power to influence the policy process, and when they agree on basic issue characteristics such as the definition of and solution to the problem. Regarding ideas, an issue is likely to get attention if described in a manner that is acceptable within the policy community [internal frame] which is typically made up of a variety of actors who have similar or competing interests around the issue of focus; and is portrayed externally in a manner that appeals to the policy-makers (leading them to, for example, allocate resources to the policy) [external frame]. Political support levels are also influenced by key features of the problem, the issue characteristics, such as: severity of the problem, the ease with which the problem can be measured and monitored, contestation around the problem, and whether there are inexpensive and evidence-based interventions available as solutions. In addition, political support levels are influenced by events and conditions surrounding the policy process, that is, the political context, which include policy windows (eg, elections and global agendas), as well as formal and informal institutions. The term “institutions” was clarified by Walt and Gilson^31^ as meaning the “formal and informal norms and rules” that make up judicial and legal institutions at the global and national governance level; and they also recognised the influence of historical factors. Finally, the outcome component was added by Walt and Gilson^31^ to examine whether an issue has been prioritised ie, is being taken seriously by national policy-makers as evidenced by authoritative decisions (such as making appropriate legislation and policies) and/or allocation of domestic resources.

###  Study Design

 The study adopted a single-case study design as the aim was to explain empirically a “contemporary phenomenon” (‘the case’) within its real-life setting, where the distinction between the phenomenon and its surrounding context is unclear.^36^ The case is *the political prioritisation of PBF in Kilifi county between 2015 and 2018. *2015-2018 was the period between the communication of PBF to the county managers in Kilifi (2015), to the point that donor funding for PBF ceased and the implementation of PBF ended in Kilifi (2018).

###  Study Setting

 The study was conducted in Kilifi county situated at the Kenyan coast.^37^ It has an estimated population of 1.5 million, of which, 48% and 52% are male and female, respectively.^37^ Kilifi county department of health (CDoH) is responsible for health service delivery across the county.^37^ Provision of healthcare services is split evenly between public and private facilities.^37^ Public health services within the county are organised around the following five levels: community health services (level one), PHC (dispensaries and health centres- levels two and three), and county referral services (levels four and five hospitals).^37^ There are four nurses and one doctor per 100 000 population in Kilifi.^37^

###  Data Collection Procedures

 Data collection procedures were developed by DW under the supervision of MO and LG. DW led the data collection. It included a review of 19 documents shown in Supplementary file 2,^27,29,39-55^ and 8 in-depth interviews with national and county actors. The specific site for data collection (Kilifi county) was selected based on two criteria: (*i*) a county where PBF was to be implemented and (*ii*) accessibility to the site (given a long standing relationship with Kilifi county managers^38^). The in-depth interviews were conducted between April and September 2020.

 The interviews were guided by a semi-structured interview guide (see Supplementary file 3) informed by the conceptual framework. They entailed online audio-recorded conversations lasting about an hour with purposively selected study participants. The decision to conduct online interviews (rather than face-to-face) was due to the coronavirus disease 2019 (COVID-19) pandemic. Participants were selected on the basis that: (1) they were either knowledgeable about PBF’s introduction and implementation in Kilifi and/or (2) were directly involved in PBF’s introduction and implementation process to Kilifi county. The first study participants were selected after an initial review of the documents, and subsequently the snowballing technique was used to identify additional participants. The participants were then recruited through telephone calls and email invitations. Four invited interviewees refused to participate for unknown reasons, and an additional two invitees noted that they felt they were not adequately involved with PBF. Nevertheless, we were able to capture the perspectives of eight national and county actors, including respondents from health and finance, and one respondent with particular knowledge of the political environment in Kilifi. We triangulated the findings with the documents reviewed.

 A range of documentary material from both national and county levels that contained information pertinent to the introduction and/or implementation of PBF in Kilifi county were reviewed, as identified in Supplementary file 2. This material was identified by interviewees, and from targeted internet searches in google, government websites and the World Bank’s results-based financing (RBF) websites.

###  Data Analysis

 The interview recordings were transcribed verbatim and the coding of both interview transcripts and documents was done manually by DW and reviewed by LG and MO. For both data sets, a framework analysis approach was adopted to provide findings and interpretations that are relevant for policy and practice.^56,57^ This involved five iterative steps: (1) familiarization by listening to the audio-recordings and reading the transcripts and documents for review; (2) developing a coding scheme by drawing upon the study’s conceptual framework; (3) reading through the transcripts and documents thoroughly and manually linking the relevant findings to the coding scheme in a deductive coding process; (4) sorting and charting the data according to the coding scheme and; (5) critical examination and interpretation of the charted data across respondents, documents and themes to generate explanations and in-depth understanding of the data.

 Our deductive codes were derived from an analytic framework of relevance to our study question, and the further application of this framework in our analysis also serves to enhance analytic rigour. To enhance the validity of the descriptions and explanations of findings, we used more than one method of data collection (interviews and document reviews) and looked for patterns of convergence in the findings by comparing the data across interviewees, and between the interviews and documents. Finally, to enhance credibility, the preliminary findings from the interviews and documents were reviewed collectively by research team members to think through analytic points, check and test assumptions made in analysis and deepen descriptions and explanations. The work was also discussed periodically and critically reviewed by two in-country health financing and governance experts, EB and BT, to reflect on the ideas emerging from the data and to identify relevant actors.

## Results

###  Summary Statement 

 National policy elites gave sustained attention to PBF as a priority for implementation across ASAL counties in the period 2015-2018. National actors had secured donor funding to support PBF implementation in ASAL counties; developed some PBF policy documents (specifically, operational manuals and guidelines); and led PBF sensitisation and training in the ASAL counties. However, the Kilifi county governor, influenced by positions taken by the county’s legal and finance team, delayed (by almost 3 years) the signing of a performance agreement with the national MoH, thereby stalling PBF implementation in Kilifi county. Ultimately, only four months of implementation at service delivery level was possible in 2018, before donor funding ended. No county level, domestic funding was allocated to PBF at any time, and no legislative actions were taken by Kilifi county to mandate the continued funding of PBF beyond the period after donor funding withdrawal as part of the county’s health financing arrangements. This failure to fund PBF within the county is a clear indication that the sub-national level did not accord this policy political priority.^23^

 A summary of the key explanations of this experience as highlighted by application of the adapted Shiffman and Smith^23,31^ political priority setting framework are shown in Table 3, and discussed in more detail below. As Table 3 highlights, the intersection between political context, intervention design and actor power was a specific explanation of this experience, alongside issues linked to each of the four elements of the Shiffman and Smith framework. All points are discussed further in Table 3.

**Table 3:** Summary of the Key Themes Through the Lens of the Adapted Shiffman and Smith^23,31^ Political Priority Setting Framework

**Framework Category**	**Findings**
Intersection between the political context, intervention design and actor power	Adoption of new constitution and devolution (change in *political context*) led to: 1. Introduction of a new PFM Act^29^ which resulted in *contestation* between national and county levels, over the initial design of the PBF policy (‘intervention design’ as an issue characteristic). This led to roughly over a year’s delay in implementing PBF, and changes to the PBF policy design. 2. The national MoH’s inability to be an *effective guiding organisation*. 3. Introduction of new county level non-health sector bureaucrats (ie, county treasury and legal team) and politicians (county governor and members of county assembly) who then played key roles in PBF’s implementation by the county beyond donor timelines. As they had not been involved in PBF’s earlier stages of inception, piloting and design and, the subsequent PBF sensitization, training and *decision-making spaces,*they had limitedunderstanding of and buy-in to PBF.
Issue characteristics	In the newly devolved context of Kilifi county, the underlying features of the problem which PBF sought to address (health worker motivation and MCH indicators) were rarely discussed outside the health sector, and were uncontested by health sector actors. However, the intervention design (the solution) was contested by the county treasury and legal team.
Ideas	The internal framing of PBF by the national MoH and CDoH may have affected the public positioning of PBF and whether/how it attracted the attention of the county political elites. Specifically: 1. The idea of ‘pay for performance’ was perceived as contradictory to the PFM Act’s^29^ planning and budgeting processes and timelines. 2. The framing of PBF as a donor-funded programme providing much-needed ‘additional funding’ for healthcare providers became interpreted as an ‘additional expense’ when it needed to be funded by the county government in the long-term following the end of World Bank PBF funding.
Actor power	The failure to mobilise key county politicians (specifically, MCAs) who have *high political power* was a challenge since their support was essential in passing relevant county legislation and approving financial allocation for PBF beyond the World Bank’s funding period.
Outcome	There was contestation within Kilifi county in the period 2015-2018 about the prioritisation of PBF. PBF implementation stopped at the end of 2018 when donor funding concluded, as no resources were allocated by the County to continue implementation and no further arrangement was made with external actors.

Abbreviations: PBF, Performance-based financing; MoH, Ministry of Health; MCAs, Members of the County Assembly; MCH, maternal and child health; PFM, public financial management; CDoH, County Department of Health.

###  Intersection Between the Political Context, Actor Power and “Intervention” Design as an Issue Characteristic 

 The findings reveal that global health policy priorities such as the ‘shift in health financing from inputs to results’ and Millennium Development Goals 4 and 5, helped frame an agenda for PBF’s introduction in Kenya.^41,44,45,50,54,55^ Thus, between October 2011 and 2013, the MoH, with financial and technical support from the World Bank, set up a PBF pilot project in one of the ASAL regions known as Samburu district (later became a county).^27,44-46,52^ An ASAL pilot district was selected as their performance in MCH indicators was reportedly worse than other regions in the country. Following its end line evaluation, the PBF pilot was considered a “success”^41,44,45,54,55^ by the MoH and the World Bank due to the improvements seen in facility management (eg, availability of infrastructure, staff trainings and regular meetings) and in some service utilisation indicators (eg, under-five child welfare clinics attendance).^27,52,53^ Therefore, it was agreed that PBF should be implemented in other ASAL regions (including Kilifi). In addition, a non-ASAL region called Migori was included as one of the PBF implementing counties because its MCH indicators were also performing poorly. Implementation was meant to commence after the pilot project, at the end of 2013. However, the national political context changed in June 2013 due to the adoption of a devolved system of governance in the country. Devolution had three significant effects on the PBF policy process in Kilifi.

 First, the changing governance context reportedly delayed the rolling out of PBF in Kilifi and other counties selected for PBF implementation for over a year. The adoption of the PFM Act^29^ introduced new PFM structures and processes that were different from the original intervention design for PBF. Pre-devolution, the HSSF mechanism was used for direct transfer of funds to facilities. According to the new PFM Act,^29^ all the funds meant for facilities would be sent to a centralised account first (the County Revenue Fund) and then distributed to facilities by the County treasury.^43^ As a result, there was contestation between the county governments and national actors (MoH, World Bank and national treasury), about whether PBF implementation should be allowed to circumvent the new PFM structures and processes (by sending funds to health facilities directly).

 “*I think that [devolution] could have been the reason why even the scale up delayed so much because the whole question was when the devolved units came into existence, there is the issue of direct funding of facilities became quite a subject matter that really took a lot of effort to try and agree on how the system that was there previously was aligning with the public finance management act twenty twelve which said the money was supposed to go in a certain way to the county, you know, through the County revenue fund and all that... So we took quite a bit of time but I think eventually – I think it was agreed – I want to say the public finance management Act really was an impediment to that process [of scaling up PBF]” *[NM01, 2020, national level actor].

**Figure 1:**
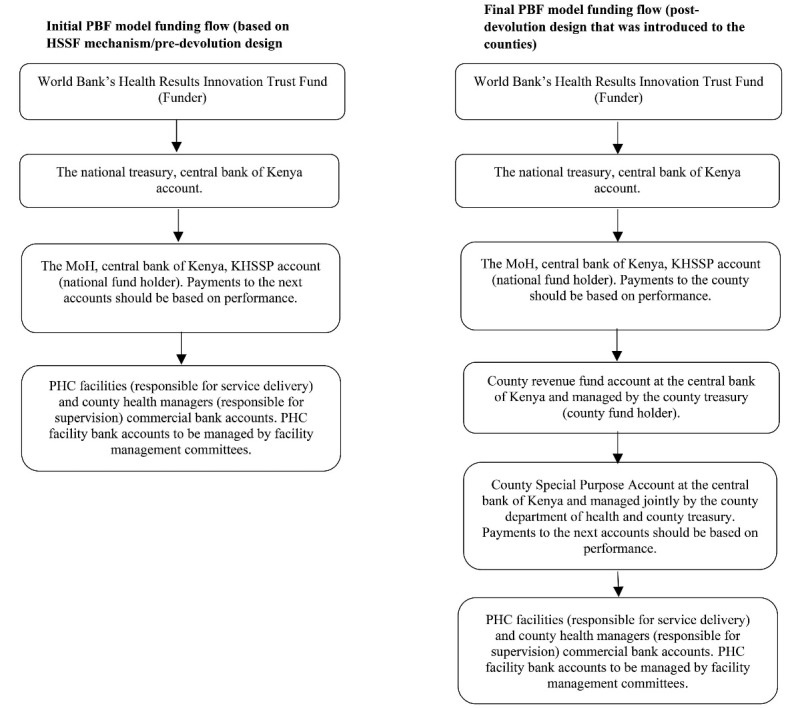
Changes in the Performance-Based Financing Funding Flow Arrangements Pre- and Post-devolution. Abbreviations: MoH, Ministry of Health; HSSF, Health Sector Service Fund; KHSSP, Kenya Health Sector Support Project; PHC, Primary healthcare.

 Eventually, the national MoH, national Treasury and World Bank agreed to make some changes to features of the PBF policy design before it was introduced to Kilifi county. For example, as illustrated in Figure 1, post-devolution, the county Treasury was assigned the role of fundholder, rather than funds being sent through the MoH direct to facility bank accounts.^41,43^ In addition, the national actors through the national MoH and national treasury instructed that the PBF funds be transferred to a ring-fenced county health special purpose account that would be jointly managed by the CDoH and county treasury.^41-43^ From the special purpose account, the PHC facilities would then be paid based on their verified performance in the incentivised indicators.^41,43,49^ In addition to Figure 1 shown here, Supplementary file 1 provides the full details of the initial PBF policy design (based on the HSSF mechanism/pre-devolution policy design) and final PBF policy design (ie, post-devolution policy design). Noteworthy, all the PBF implementing counties were required to first sign a performance agreement between themselves and the MoH as a commitment to implement PBF as per the final policy design.^41,43^ This was one of the pre-conditions for the counties to access and spend the PBF funds.

**Figure 2:**
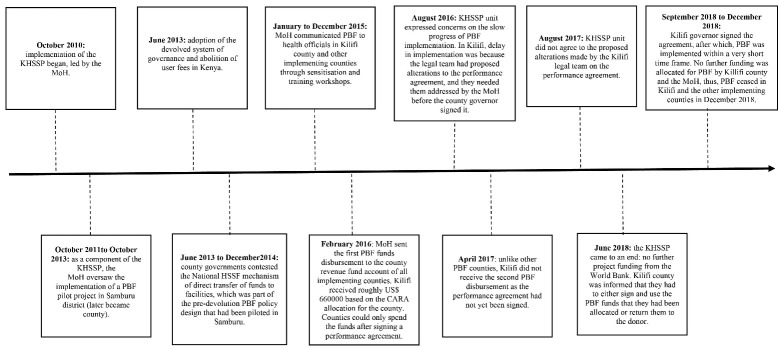
Timeline of Key Events in the Performance-Based Financing Policy Process in Kilifi County. Abbreviations: PBF, Performance-based financing; MoH, Ministry of Health; HSSF, Health Sector Service Fund; KHSSP, Kenya Health Sector Support Project; CARA, County Allocation of Revenue Act.

 Second, devolution impacted on actor power dynamics, undermining the national MoH’s ability to lead and be an *effective guiding organisation*for PBF by reducing its influence at the county level. Pre-devolution, the MoH in consultation with the World Bank had taken the lead role in the design, piloting and eventual roll out of PBF in the country^40^ (see Figure 2). After devolution, however, responsibility for health service delivery, including the management of human resources and PHC facilities, was assigned to county governments. Neither the county government nor governor were answerable to the MoH^58^ and both had very high *political power*at the county level.^59^

 “*The counties would say we are now devolved, we are the ones responsible for this service delivery, you cannot be the one running this programme, you should give us the [PBF] money … What is the ministry [of health] doing with a small facility in Mandera or in Kilifi or wherever? That business should be left to the county governments because, after all, health is devolved, you know, there was always the thing of schedule four [of the constitution], health is fully devolved. The work of the ministry should be policy and strategic guidance not operationalizing things at the county” ***[**NMO1, 2020, national level actor].

 Third, devolution introduced new county level bureaucrats outside the health sector (ie, county treasury and county legal team) and politicians (county governor and members of county assembly) into the PBF policy process.^50^ These actors had key roles to play in PBF’s acceptance and uptake as part of the county’s health financing arrangements. However, they had not been adequately involved in the earlier stages of inception, piloting and design or the subsequent sensitisation and trainings. The latter were directed at the county health managers, health workers and health facility management committees.^44-46^ In addition, the introduction of PBF was experienced as a ‘top-down’ process as the national MoH provided instructions for its implementation through the CDoH. These factors limited the understanding of and buy-in for PBF by the county non-health sector bureaucrats and governor, and the treasury and legal team, in particular, who saw PBF as a threat to their newly acquired county level functions and *bureaucratic powers*.^59^ They were particularly challenged by key design features, especially the opening of the special purpose account and the county governor needing to also sign a performance agreement. The county treasury, responsible for the control and management of all public funds held in the county revenue fund (*actor interest*), contested the need to set up a separate ring-fenced county health special purpose account for PBF, and jointly manage it with the CDoH (*intervention design*). Similarly, the county legal team, responsible for advising the county government and governor on all legal matters, including those related to their relations with the national government (*actor interest*), raised questions about the need for the county governor to sign the performance agreement between the Kilifi county government and MoH, developed without county involvement (*intervention design*). These design features challenged the newly acquired “*bureaucratic power*” of county actors, including their control of county public funds.^59^

 “*The challenge was between the legal team not accepting the fact that there are funds that have been brought and they came with a contract, the performance contract. And it was to be signed by the governor for us to be able to implement. Also, for finance, being not able to understand how this programme was being implemented. You see, because RBF [PBF] implementation was a bit different. And as much as money was supposed to come through the county revenue fund, still this money was supposed to end up in what we call a special purpose account which was to be opened” *[SBM003, 2020, county level actor].

 Two county participants suggested two other reasons for the contestation around PBF design features. They noted that: (1) county level actors did not have established, historical working relations and trust with those making the key decisions on PBF’s introduction and implementation, the donors, the national treasury and the national MoH (*historical dimension*), and (2) county level attempts to seek clarity on the contents and duration of the performance agreement between the county and national MoH, by suggesting some alterations to the performance agreement (in August 2016), were rejected by the KHSSP unit on the grounds that the national actors had already finalised the agreement.

 “*The fact that maybe they [national treasury/ MoH ] have been able to deal with these donor funds many times made it easier for them; you know it was not the first time… But for us [at the county] it now became a challenge because the new people that we had [as part of devolution structures] you don’t know from which background that these people were coming from; maybe they were in private institutions, then you come to government and you have to learn again” *[SBM002, 2020, county level actor].

 “*But now for the Ministry of Health, this [ ie, performance agreement] was a document that they had developed for all the [PBF] counties. So, they did not see any reason for them to change it” *[SBM003, 2020, county level actor].

 County actors outside the health sector also interpreted the timing of PBF’s introduction in the *newly devolved context* as having political motives. Specifically, they judged that the incumbent national government sought to use an “innovative” health sector funding scheme to win political votes for the upcoming 2017 national and county elections. The Kilifi’s elected county governor was a key member of the main opposition political party.

 “*Now the legal advisor…. [Because]at the back of her mind she knew that health is devolved. so, she said now, why is the national government coming in with these innovations this time? then that is when now somehow the Jubilee and ODM issues came in because you know our governor is from ODM and the president is in Jubilee party. So, you see now people started associating these things politically which was not right. Yeah” *[SBM004, 2020 county level actor].

 Further, despite members of the county assembly (MCAs) having high *political power* at the county level,^59^ they were not mobilised to support PBF.^50^ Yet, their advocacy and support was essential in determining whether any legislative actions would be taken, or financial allocation to PBF made, at the county level in the long-term. Section 104 of the PFM Act^29^ stipulates that no public funds shall be appropriated outside a county’s planning and budgeting framework approved by the county assembly. One participant reflected on how the failure to create PBF awareness among MCAs denied him the opportunity to urge them to support its implementation post donor funding timelines:

 “*…. During my time at least [as a senior bureaucrat within Kilifi CDoH ] then I do not remember whether there was a Member of the County Assembly who came to say okay I heard about this [PBF] in this country so maybe why don’t we start implementing, I never heard. I would probably tell them there is these kinds of funding mechanism, why don’t we introduce, you know” *[CM001, 2020, county level actor].

###  Issues With the Understanding and Framing of Performance-Based Financing 

 Key actor groups (ie, those involved in PBF implementation in Kilifi county) included the World Bank, MoH and National Treasury at the national level; and the CDoH, the county treasury, the county legal team and the county governor at the county level. Amongst these actor groups, the national level actors and the CDoH supported the implementation of PBF within the county, whereas the county treasury, legal team and governor had reservations about PBF, specifically around the setting up of a special purpose account and signing of an established agreement, as discussed earlier. Furthermore, these latter actors were drawn to the issues around PBF’s design features, and rarely discussed the underlying features of the problem which PBF was addressing (*issue characteristics*), possibly limiting their understanding of the potential role that PBF could play in the county. However, even amongst the ‘domestic’ actor groups who supported PBF, two key issues in the understanding and framing of PBF (ie, the internal frame) may have affected how PBF was portrayed publicly thereby affecting its attraction to the political elites at the county level.

 First, the idea of ‘pay for performance’ was perceived to be contradictory to the PFM Act’s^29,51^ planning and budgeting processes and timelines. According to the law, the amount of funds that could be sent to a county was not supposed to exceed those that had been pre-estimated in the County Allocation of Revenue Act (CARA)^39^ that guides government resource allocation to counties. However, PBF funding was intended to be linked to performance and was likely to exceed the estimated CARA annual allocation to Kilifi. In practice, therefore, the amount of PBF funds sent to Kilifi was reportedly not based on actual quarterly performance but rather on the MoH ‘estimates’ determined before the financial year began.^51^ For example, even before Kilifi county signed the agreement and started implementing PBF, in February 2016, it received the first PBF disbursement of about US$ 660 000 from the World Bank, based on the County’s CARA allocation rather than performance linked to the PBF incentivised indicators. This approach defied the internal logic of PBF (ie, payments linked to efforts/performance) and perhaps also underlay contestation around the design of PBF (*intervention design*).

 “*So the challenge was this PBF money because you know if it is based on performance you cannot be absolutely sure in advance how much county A is going to make, isn’t it? Because it will depend on the deliveries, the children immunized and all that. So basically when ministry [of health] is submitting the estimates for a given year that this particular money let’s say it’s under PBF for county A or B, so chances are that eventually what is disbursed to the county is more or less than what is captured in the CARA. But if it is more then how do you account for that difference? Does it mean that the estimates had to be revised or what exactly? They [at the National Treasury] would not just approve that money to be channelled to the counties. So, I think those are the issues just around the PFM Act and I think they were quite complicated. *[NM001, 2020, national level actor].

 Second, the health sector actors (MoH and CDoH) understood and framed PBF as a donor-funded programme providing much-needed ‘additional funding’ for healthcare providers (health managers, health workers and PHC facilities) for a defined time period, after which the donor funding would end like other programmes. This understanding and framing of PBF was influenced by the perception that the PHC facilities had always been underfunded. However, this framing of PBF did not appear to be politically attractive outside the health sector at the county level. Instead, PBF was seen at county level as a potential future ‘additional expense’ should they decide to carry on implementing PBF following donor funding withdrawal. One participant noted this might be due to a lack of understanding of the ‘problem’ and ‘solution’ that PBF offered, as shown here:

 “*It would be difficult for PBF initiatives to really take off and to be funded [by the government]. The question is, if I have paid the health worker, I have provided the infrastructure, I have provided the essential medicines, why am I paying extra for PBF? You know, as policy-makers, that’s what they would ask. So, the question is how do you convince them of the value of this because I believe that is the link, that is the gap that is missing” *[NM001, 2020, national level actor].

 Together these factors contributed to the three-year (2015-2018) delay in the signing of the performance agreement by the Kilifi county governor (see Figure 2). As the performance agreement was not signed, Kilifi county could not spend the first disbursement of PBF funds at their disposal. Subsequently, in April 2017, when other implementing counties were receiving their second PBF disbursement after having spent their first disbursement, Kilifi county missed out on a second disbursement.^47^ The governor’s eventual decision to sign the performance agreement was perceived to have been motivated by the fact that the county was informed that it had to either sign and use the first disbursement of PBF funds (ie, the US$ 660 000 mentioned earlier) by the end of the KHSSP, or return the funds. Following the signing, PBF was implemented at service delivery level over a span of about four months between September and December 2018. This required verification of facilities’ performance in the previous quarter, and the payment of performance bonuses to the facilities, health workers and health managers. There was reportedly no subsequent progression of PBF related activities in Kilifi and PBF was not taken up by the county beyond the World Bank’s KHSSP funding timelines.

 “*It really did not go according to plan because it was to be implemented in that financial year 16/17 but it didn’t, it didn’t take off. It was implemented in financial year 18/19. Then it was more of compliance because we had gotten to a point where these monies had to go to the facilities or the county government refunds the money to World Bank... it was done in about a quarter, so four, three months”* [SBM002, 2020, county level actor].

Figure 2, finally, shows a summary of the timeline of PBF related events and activities as outlined above, with a specific focus on those related to Kilifi county. It shows how the PBF policy process led by the MoH encountered contestation over key design features of the PBF intervention upon devolution, which subsequently delayed the signing of the PBF performance agreement and its implementation in Kilifi.

## Discussion

 To the best of our knowledge this study is the first to examine the political prioritisation of PBF at a sub-national level, an analysis appropriate in Kenya because of its devolved governance structure. In this work we also aimed to test, at the sub-national level, the utility of applying the adapted Shiffman and Smith^23,31^ political priority setting framework. Ultimately, we identified an interacting set of factors that offered critical explanations of why PBF was not sustained as a high priority issue for implementation at the service delivery level and was not funded within Kilifi county post donor funding. We first discuss the intersection between the political context and actor power. Second, we discuss the challenges that decentralisation reforms pose to the implementation of health programmes. Third, we discuss misalignment between the ideas/framing of PBF and the political context. Fourth, we discuss key issue characteristics linked to the intervention design.

 The existing literature on national level political prioritisation of health sector reforms has shown the importance of national level bureaucratic (such as national MoH) and political (such as national Ministers of Health and Presidents) actors in effectively guiding and leading the adoption of health sector reforms. For example, in Ghana^60^ and Ethiopia,^61^ the national MoH led by their Ministers of Health played key roles in effectively managing stakeholders’ interests and advancing national level health insurance policy agendas. Likewise, in both Armenia and Rwanda, “strong political and technical leadership” by the national MoH was part of the reason for PBF’s evolution from a programme to a national policy.^6^ The ability of these national level actors to lead their respective countries’ political prioritisation processes effectively was arguably because of their relatively more centralised systems of governance. Our findings show that in highly devolved systems, policy prioritisation and leadership by national level actors does not guarantee successful policy prioritisation and uptake by sub-national actors.

 Decentralisation reforms usually have laudable goals (such as promoting community involvement, accountability, efficiency, and equity in resource management), which aim to improve health service delivery and public health. However, in practice, these goals are rarely achieved due to technical^28,62^ and/ or political obstacles.^63-65^ In our study, devolution resulted in a significant change in context for the policy of focus as it altered power relationships between the national and sub-national level. Because of the new political power at the sub-national level, PBF implementation at service delivery level was not prioritised. Similarly, in Brazil decentralisation of fiscal and administrative decision-making capacities to state and municipal governments affected the performance of the national HIV/AIDs programme, formerly run by the federal MoH.^63^ This was linked to the newly weakened position of the federal MoH in programme oversight, and the empowering of conservative local elites who had little interest in supporting the funding of some of the programme activities eg, campaigns aimed at commercial sex workers.^63^ In the early 1990s in the Philippines, although the family planning programme was one of the main national health sector priorities, upon devolution, a provincial governor stopped the delivery of family planning services in his province due to religious convictions.^64^

 Recently, the influence of ideas, rather than only actors’ interests, over the political prioritisation of policy options at the national level has gained recognition.^66^ For example, studies on agenda setting for community based health insurance policies in Ethiopia^61^ and Rwanda^66^ noted that these policy reforms were adopted and promoted nationally due to their alignment with the ruling political party’s ideologies, centred on ‘self- reliance’ and ‘visible’ community participation. However, our findings at the sub-national level in Kilifi suggest that lack of alignment between the ideas underpinning a reform and the political context, undermines reform’s relevance for actors. In Kilifi for example, there seemed to be conflict between the globally advocated idea of ‘pay for performance’ and the pre-existing centralised and rigid approaches to PFM leading actors to contest the programme design. In addition, the idea that PBF could be understood as ‘additional funding’ was contested as county actors felt it would be an ‘additional expense’ to the county government once the World Bank stopped funding PBF. As a result, PBF was not sustained as a high priority issue at county level despite the initial availability of donor funds as, when these were spent, no further allocations to the programme were made by Kilifi County. As also more widely identified, the use of donor funds to support a policy does not necessarily imply that government has prioritised it.^6^ Given that donors mostly set the priorities for how their money will be spent, the ways in which national – or, in this case, sub-national, governments allocate their domestic resources is a strong indicator of political prioritisation at the national level.^31^

 Beyond the explanations of PBF priority-setting in Kilifi county, this study offers insights about “issue” characteristics as a key determinant of political priority, as emphasised in the Shiffman and Smith^23^ framework. Our findings at a sub-national, reveal limited engagement between national and county level actors over the characteristics of the ‘problem’ that PBF was meant to address in health facilities. This may have been because the sub-national actors became involved in PBF’s scaling up at a late stage of decision-making. At this late stage, there may have been less motivation for sub-national actors to examine and scrutinise the features of the problem to which a solution had already being decided; key actors also then included non-health sector actors at the county level. The intervention design features were, however, a key determinant of political priority within the new devolved governance context. Issue characteristics in the Shiffman and Smith framework^23^ include some consideration of intervention design, but wider literature more clearly points to their importance. Grindle and Thomas,^67^ specifically, describe these intervention design features as “policy characteristics” influencing the acceptability of policies. Key characteristics are: the distribution of costs and benefits associated with implementation across policy actors and society; the technical and administrative complexity of the reform; and the duration needed for visible impact. Crichton,^68^ in another Kenyan study, meanwhile, argues that the prioritisation and implementation of a family planning policy was affected by these policy characteristics (including intense administrative and technical requirements, and opposition linked to cultural and religious sensitivity on contraceptive use). In addition, and similar to our findings at the sub-national level, concerns and contestation over intervention design (the solution) appears to be a key feature of national level political prioritisation of health financing reforms in other countries. In Ghana, there were delays in the agenda setting process due to contestation over the design of the National Health Insurance Scheme between the Minister of Health who wanted a single-payer social insurance and the National Health Insurance Scheme task force members who wanted multi-payer mutual health organisations.^60^ In South Africa, the introduction of a National Health Insurance was delayed in part due to political disputes and contestation, including by provincial governments who seemingly felt that the proposed design of the National Health Insurance threatened their roles and powers.^69^

 We make three final observations in testing the Shiffman and Smith framework. First, we acknowledge that Walt and Gilson^31^ highlighted the allocation of domestic resources as evidence of an ‘outcome’ of political prioritisation at the national level. We argue, based on this study, that the allocation of domestic resources at the sub-national level is also important as an indicator of political priority in a devolved governance context. Second, as the Shiffman and Smith^23^ political priority setting framework appears to be currently rooted in a central/national government perspective, it does not allow for the multiple levels of political prioritisation required in devolved governance settings. Therefore, we propose that the formal and informal institutions at ‘devolved levels of governance’ should be considered as part of the governance structure element in political context to make the framework more applicable at the sub-national level. It would also be important to include the range of health and non-health sector sub-national actors as part of the leadership element in actor power. Finally, we argue that ‘issues characteristics’ can be usefully expanded to include policy characteristics,^67^ as well to broaden the scope of issues under study.

###  Study Limitations

 We acknowledge four key limitations. One, this study conducted research in only one county in Kenya, and experience may have been different in other PBF implementing counties. This work was initiated in Kilifi county given long-standing relationships with county managers – relationships that also enabled virtual interviewing during the period of COVID-19 restrictions. It was simply not possible to conduct interviews in other counties; neither were we able to source documentation that offered insights on implementation in other counties. Two, interviews were conducted online due to COVID-19 research regulations. Challenges related to conducting online audio interviews included lack of non-verbal cues and network connection problems. Three, it was not possible to interview some key actors mentioned by respondents, such as the county treasury, legal team and county governor, whose views would have enriched our study. Although efforts were made to reach these actors, our initial requests for an interview were either unanswered or refused as the actors judged that they had had minimal involvement in PBF. Thus, our study is limited by the small number of interviews conducted. However, the respondents who were interviewed were themselves among those critical to the process we examined and had relevant experience and views. In addition, in our analysis we draw both on these interviews and a wide set of documents, some of which captured the views of key actors we were not able to interview. Whilst saturation may not have been reached through the interviews alone, this combined source material provided an array of experience and perspectives and a rich foundation for our analysis. Four, some of the study participants struggled to remember retrospectively the details and/or exact chronology of events of PBF introduction. This limitation was partly addressed through triangulation with the reviewed documents.

## Conclusion

 This study contributes to theory building by empirically testing the Shiffman and Smith^23^ political priority setting framework with adaptations by Walt and Gilson.^31^ We argue that the framework can be useful at the sub-national level and have recommended additions.

 The study also contributes to the limited but growing body of literature on the politics of policy processes.^70^ The framework was helpful in identifying and understanding the key interacting factors that led to contestation over the prioritisation of PBF at the County level in Kenya between 2015-2018.

 For policy-makers, advocates or researchers aiming to influence the political prioritization of health reforms in highly devolved contexts, adequate, early involvement and leadership from sub-national bureaucratic and political actors, both in health and beyond the health sector, is important for policy uptake. In addition, the centrality of ideas and the ways in which ideas resonate (or not) with actors is key to uptake. Finally, the political context including political and bureaucratic power at different levels of government are crucial features that influence the acceptability of reform and ultimately political prioritisation at sub-national level in devolved contexts.

## Acknowledgements

 This work would not have been possible without the support and cooperation of the Kilifi county and national level actors.

## Ethical issues

 This study obtained ethical approval from the University of Cape Town Human Research Ethics Committee (Reference number: 086/2020) and the KEMRI Scientific and Ethics Review Committee (Reference number: KEMRI/SSC/2795) in Kenya. In addition, authorisation for data collection in the county was obtained from the Kilifi County Department of Health and from all the study participants.

## Competing interests

 Authors declare that they have no competing interests.

## Authors’ contributions

 DW, LG, and MO were involved in the conceptualisation of the study and protocol development. DW conducted data collection, conducted analysis and drafted the original manuscript. All authors were involved in reviewing the draft, final analysis and editing. All authors read and approved the final manuscript for publication.

## Funding

 This work was funded by the International Masters Fellowship awarded to DW with funds from the Wellcome Trust (grant reference: 214624/Z/18/Z). DW, BT and EB are members of the KEMRI-Wellcome Trust Research Programme in Kenya that is supported by a core grant (grant reference: 203077/Z/16/Z) from Wellcome Trust.

## Supplementary files


Supplementary file 1. Similarities and Differences Between the Initial PBF Policy Design (ie, Based on the HSSF Mechanisms/Pre-devolution Policy Design) and the Final PBF Policy Design (ie, Post-devolution Policy Design).

Supplementary file 2. Overview of Documents Included in the Case Study (n = 19).

Supplementary file 3. Interview Guide.

## References

[1] World Health Organization (WHO). Health Financing for Universal Coverage. https://www.who.int/health_financing/topics/performance-based-financing/universal-health-coverage/en/. Published 2019.

[2] Eichler R, Auxila P, Antoine U, Desmangles B. Performance-Based Incentives for Health: Six Years of Results from Supply-Side Programs in Haiti. Washington, DC: Centre for Global Development; 2007.

[3] World Health Organization (WHO). Aid Effectiveness and Health. WHO; 2007.

[4] Meessen B, Soucat A, Sekabaraga C (2011). Performance-based financing: just a donor fad or a catalyst towards comprehensive health-care reform?. Bull World Health Organ.

[5] Morgan L. Results-Based Financing for Health (RBF): What’s All the Fuss About? 2014. https://www.rbfhealth.org/resource/results-based-financing-health-rbf-what%E2%80%99s-all-fuss-about.

[6] Shroff ZC, Bigdeli M, Meessen B (2017). From scheme to system (part 2): findings from ten countries on the policy evolution of results-based financing in health systems. Health Syst Reform.

[7] Gautier L, De Allegri M, Ridde V (2019). How is the discourse of performance-based financing shaped at the global level? A poststructural analysis. Global Health.

[8] Oxman AD, Fretheim A (2009). Can paying for results help to achieve the Millennium Development Goals? Overview of the effectiveness of results-based financing. J Evid Based Med.

[9] De Allegri M, Bertone MP, McMahon S, Mounpe Chare I, Robyn PJ (2018). Unraveling PBF effects beyond impact evaluation: results from a qualitative study in Cameroon. BMJ Glob Health.

[10] Soucat A, Dale E, Mathauer I, Kutzin J (2017). Pay-for-performance debate: not seeing the forest for the trees. Health Syst Reform.

[11] Waithaka D, Cashin C, Barasa E (2022). Is performance-based financing a pathway to strategic purchasing in sub-Saharan Africa? A synthesis of the evidence. Health Syst Reform.

[12] Paul E, Renmans D (2018). Performance-based financing in the heath sector in low- and middle-income countries: is there anything whereof it may be said, see, this is new?. Int J Health Plann Manage.

[13] Paul E, Albert L, Bisala BN (2018). Performance-based financing in low-income and middle-income countries: isn’t it time for a rethink?. BMJ Glob Health.

[14] Antony M, Bertone MP, Barthes O (2017). Exploring implementation practices in results-based financing: the case of the verification in Benin. BMC Health Serv Res.

[15] Ridde V, Yaogo M, Zongo S, Somé PA, Turcotte-Tremblay AM (2018). Twelve months of implementation of health care performance-based financing in Burkina Faso: a qualitative multiple case study. Int J Health Plann Manage.

[16] Zitti T, Gautier L, Coulibaly A, Ridde V (2019). Stakeholder perceptions and context of the implementation of performance-based financing in district hospitals in Mali. Int J Health Policy Manag.

[17] Gautier L. From Ideas to Policymaking: The Political Economy of the Diffusion of Performance-Based Financing at the Global, Continental, and National Levels [thesis]. Université de Montréal; 2019.

[18] Barnes A, Brown GW, Harman S (2015). Locating health diplomacy through African negotiations on performance-based funding in global health. Journal of Health Diplomacy.

[19] Kiendrébéogo JA, Shroff ZC, Berthé A, Yonli L, Béchir M, Meessen B (2017). Why performance-based financing in Chad failed to emerge on the national policy agenda. Health Syst Reform.

[20] Chimhutu V, Tjomsland M, Songstad NG, Mrisho M, Moland KM (2015). Introducing payment for performance in the health sector of Tanzania- the policy process. Global Health.

[21] Sieleunou I, Turcotte-Tremblay AM, Fotso JT (2017). Setting performance-based financing in the health sector agenda: a case study in Cameroon. Global Health.

[22] Government of Kenya (GoK). Constitution of Kenya. National Council for Law; 2010.

[23] Shiffman J, Smith S (2007). Generation of political priority for global health initiatives: a framework and case study of maternal mortality. Lancet.

[24] World Bank. The KHSSP implementation completion and results report. World Bank; 2019.

[25] Waweru E, Goodman C, Kedenge S, Tsofa B, Molyneux S (2016). Tracking implementation and (un)intended consequences: a process evaluation of an innovative peripheral health facility financing mechanism in Kenya. Health Policy Plan.

[26] Opwora A, Kabare M, Molyneux S, Goodman C (2010). Direct facility funding as a response to user fee reduction: implementation and perceived impact among Kenyan health centres and dispensaries. Health Policy Plan.

[27] Ministry of Health (MoH). Results Based Financing (RBF) Scale Up 2014-2016: Draft 1 of the Operational Manual. MoH; 2014.

[28] Tsofa B, Molyneux S, Gilson L, Goodman C (2017). How does decentralisation affect health sector planning and financial management? a case study of early effects of devolution in Kilifi County, Kenya. Int J Equity Health.

[29] Government of Kenya (GoK). Public Financial Management Act, 2012. GoK; 2012.

[30] Barasa EW, Manyara AM, Molyneux S, Tsofa B (2017). Recentralization within decentralization: county hospital autonomy under devolution in Kenya. PLoS One.

[31] Walt G, Gilson L (2014). Can frameworks inform knowledge about health policy processes? Reviewing health policy papers on agenda setting and testing them against a specific priority-setting framework. Health Policy Plan.

[32] Tomlinson M, Lund C (2012). Why does mental health not get the attention it deserves? An application of the Shiffman and Smith framework. PLoS Med.

[33] Shawar YR, Shiffman J, Spiegel DA (2015). Generation of political priority for global surgery: a qualitative policy analysis. Lancet Glob Health.

[34] Prata N, Summer A (2015). Assessing political priority for reproductive health in Ethiopia. Reprod Health Matters.

[35] Daire J, Kloster MO, Storeng KT (2018). Political priority for abortion law reform in Malawi: transnational and national influences. Health Hum Rights.

[36] Yin RK. Case Study Research: Design and Methods. 5th ed. Thousand Oaks, CA: ‎SAGE Publications; 2014.

[37] County Government of Kilifi. Kilifi County Integrated Development Plan 2018-2022. Kilifi: County Government of Kilifi; 2018.

[38] Barasa E, Boga M, Kagwanja N (2020). Learning sites for health system governance in Kenya and South Africa: reflecting on our experience. Health Res Policy Syst.

[39] National Treasury. County Allocation of Revenue Act (CARA) of 2015. National Treasury of Kenya; 2015.

[40] GoK. Financing agreement between International Development Agency and the republic of Kenya. GoK; 2014.

[41] MoH. Results Based Financing (RBF) Scale Up 2015-2018: Final draft of the Operational Manual. MoH; 2017.

[42] National Treasury. National guidelines on the transfer of conditional grants (such as PBF) to county governments. Nairobi: In: National Treasury; 2015.

[43] MoH. Kilifi county financial guidelines on disbursement, use and reporting PBF. Nairobi: MoH; 2017.

[44] MoH. Capacity building for PBF scale up: invitation to facilitate as PBF master trainer of trainees (TOT). Nairobi: MoH; 2015.

[45] MoH. Capacity building for PBF scale up: Nominees for PBF trainer of trainees (ToT) workshop 14th-18th September at the Kenya School of Government, Nairobi. Nairobi: MoH; 2015.

[46] MoH. Capacity building for PBF scale up: Cascading PBF training in the counties. Nairobi: MoH; 2016.

[47] MoH. PBF Progress Update for the Financial Year 2015/2016. MoH; 2016.

[48] MoH. PBF procurement guidelines for essential equipment. MoH; 2017.

[49] CDoH. Kilifi sub-county health managers appointment letters to the Joint verification Team. Kilifi: CDoH; 2018.

[50] Alliance for Health Policy and Systems Research (AHPSR). The Piloting and Scaling Up of Performance-Based Financing (PBF) in Healthcare in a Devolved Governance System: Experiences from Kenya Between July 2011 and May 2015. AHPSR; 2015.

[51] World Bank. Implementation completion and results report: The Kenya Health Sector Support Project (KHSSP). World Bank; 2019.

[52] Obare F, Bellows B. Technical Assessment of the Performance-Based Finance Samburu Pilot Program in Kenya. Nairobi: Population Council; 2014.

[53] Population Council. Evaluation of Performance-Based Finance (PBF) Pilot in Samburu County, Kenya-Qualitative Research Findings. Population Council; 2013.

[54] World Bank Group. RBF Health Kenya. 2014. https://www.rbfhealth.org/rbfhealth/country/kenya.

[55] The Standard- Health. Kenya receives 2.5 billion Kenyan Shillings to improve healthcare. 2016.

[56] Green J. Qualitative Methods for Health Research. 3rd ed. Los Angeles: SAGE Publications; 2014.

[57] Bryman A. Analyzing Qualitative Data. New York: Routledge; 1993.

[58] Nxumalo N, Gilson L, Goudge J (2018). Accountability mechanisms and the value of relationships: experiences of front-line managers at subnational level in Kenya and South Africa. BMJ Glob Health.

[59] Sriram V, Topp SM, Schaaf M (2018). 10 best resources on power in health policy and systems in low- and middle-income countries. Health Policy Plan.

[60] Agyepong IA, Adjei S (2008). Public social policy development and implementation: a case study of the Ghana National Health Insurance scheme. Health Policy Plan.

[61] Lavers T (2019). Towards universal health coverage in Ethiopia’s ‘developmental state’? The political drivers of health insurance. Soc Sci Med.

[62] Tsofa B, Goodman C, Gilson L, Molyneux S (2017). Devolution and its effects on health workforce and commodities management–early implementation experiences in Kilifi County, Kenya. Int J Equity Health.

[63] Frasca T, Fauré YA, Atlani-Duault L (2018). Decentralisation of Brazil’s HIV/AIDS programme: intended and unintended consequences. Glob Public Health.

[64] Kolehmainen-Aitken RL, Newbrander WC. Decentralizing the Management of Health and Family Planning Programs: Lessons from FPMD. FPMD Project, Management Sciences for Health; 1997.

[65] Lewis BD (2005). Indonesian local government spending, taxing and saving: an explanation of pre- and post-decentralization fiscal outcomes. Asian Econ J.

[66] Chemouni B (2018). The political path to universal health coverage: power, ideas and community-based health insurance in Rwanda. World Dev.

[67] Grindle M, Thomas J. Public Choices and Policy Change: The Political Economy of Reform in Developing Countries. Johns Hopkins University Press; 1991.

[68] Crichton J (2008). Changing fortunes: analysis of fluctuating policy space for family planning in Kenya. Health Policy Plan.

[69] Gilson L (2019). Reflections from South Africa on the value and application of a political economy lens for health financing reform. Health Syst Reform.

[70] Gilson L, Orgill M, Shroff ZC. A Health Policy Analysis Reader: The Politics of Policy Change in Low- and Middle-Income Countries. World Health Organization; 2018.

