# Supplementary material for: Political Prioritisation for Performance-Based Financing at the County Level in Kenya: 2015 to 2018
Source: Int J Health Policy Manag. 2023 Feb 14;12:6909. doi: 10.34172/ijhpm.2023.6909 (PMC10125155; doi:10.34172/ijhpm.2023.6909)
Supplement: Supplementary file 1 — Similarities and Differences Between the Initial PBF Policy Design (ie, Based on the HSSF Mechanisms/Pre-devolution Policy Design) and the Final PBF Policy Design (ie, Post-devolution Policy Design). [file ijhpm-12-6909-s001.pdf]

Article title: Political Prioritisation for Performance-Based Financing at the County Level in Kenya: 2015 to 2018

Journal name: International Journal of Health Policy and Management (IJHPM)

Authors' information: Dennis Waithaka[1]*, Lucy Gilson[2,3], Edwine Barasa[1,4], Benjamin Tsofa[5,4], Marsha Orgill[2]

[1]Health Economics Research Unit, KEMRI-Wellcome Trust Research Programme, Nairobi, Kenya.

[2]Health Policy and Systems Division, School of Public Health and Family Medicine, University of Cape Town, Cape Town, South Africa.

[3]Department of Global Health and Development, Faculty of Public Health and Policy, London School of Hygiene and Tropical Medicine, London, UK.

[4]Centre for Tropical Medicine and Global Health, Nuffield Department of Medicine, University of Oxford, Oxford, UK.

[5]Health Systems Research Group, KEMRI-Wellcome Trust Research Programme, Kilifi, Kenya.

(*Corresponding author: DWaithaka@kemri-wellcome.org)

Supplementary file 1. Supplementary file 1. Similarities and Differences Between the Initial PBF Policy Design (ie, Based on the HSSF Mechanisms/Pre-devolution Policy Design) and the Final PBF Policy Design (ie, Post-devolution Policy Design).

KEY FEATURES OF KENYAN PBF	INITIAL PBF POLICY DESIGN (SOURCE: 2014 PBF OPERATIONAL MANUAL)	FINAL PBF POLICY DESIGN (SOURCE: 2017 PBF OPERATIONAL MANUAL)
Funder	World Bank	World Bank
Stated goal	To improve health outcomes for women and children and thus contribute to the achievement of the Millennium Development Goals (MDGs) four and five.	To improve health outcomes for women and children and thus contribute to the achievement of the Millennium Development Goals (MDGs) four and five.
Scale of implementation	20 Arid and Semi-Arid Land (ASAL) counties (henceforth referred as the PBF counties).	20 Arid and Semi-Arid Land counties (ASAL) **plus Migori county (a non-ASAL county).**
Incentivised services/activities	-10 quantity indicators mainly drawn from maternal and child health (MCH) services. This comprised of: child welfare services, antenatal care services, skilled deliveries, family planning services, immunisation services, new outpatient services, cervical cancer screening, and HIV counselling, testing and treatment services.<br><br>-13 broad quality assessment areas (such as general management, availability of drugs, health management information systems, hygiene and waste management).	-10 quantity indicators mainly drawn from maternal and child health (MCH) services. This comprised of: child welfare services, antenatal care services, skilled deliveries, family planning services, immunisation services, new outpatient services, cervical cancer screening, and HIV counselling, testing and treatment services.

	- Integrated supportive supervision by the county health management team (CHMT).	-13 broad quality assessment areas (such as general management, availability of drugs, health management information systems, hygiene and waste management).<br><br>- Integrated supportive supervision by the county health management team (CHMT).
Who is incentivized and how are the incentives split	-Incentives for primary health care providers (60% salaries top-up, 40% facility investment).<br><br>-Incentives for the CHMT.	-Incentives for primary health care providers (60% salaries top-up, 40% facility investment).<br><br>-Incentives for the CHMT.
Schedule of incentive disbursement	Quarterly (after every three months) performance payments.	Quarterly (after every three months) performance payments.
ASSIGNMENT OF PBF FUNCTIONS ACROSS NATIONAL AND COUNTY GOVERNMENT ENTITIES:		
1. Regulator- responsible for overall policy guidance including the development of the PBF implementation guidelines and, overseeing its implementation in the counties.	MoH with the support of a PBF technical working group comprising of the World Bank, MoH, County Department of Health and key partners.	MoH, with the support of a PBF technical working group comprising of the World Bank, MoH, County Department of Health and key partners.
2. National and county fundholder- responsible for receiving the PBF funds from the donor and ensuring that the financial management regulations and reporting requirements were adhered to at national and county levels respectively.	National fundholder: HSSF unit within the MoH (makes quarterly performance-based payments to the bank accounts of the PHC facilities and the CHMT).<br><br>**County fundholder:** None.	National fundholder: HSSF unit (name was changed to the KHSSP unit) within the MoH (makes quarterly performance-based transfers of PBF funds to the county fund holder).<br><br>**County fundholder:** county treasury (makes transfer of the PBF funds to the purchaser).
**3.** Purchaser- responsible for paying the PHC	**County PBF steering committee** (chaired by the CEC-Health official and the Chief Officer of Health as the secretary and other members to include the	**County Department of Health (CDoH)** was assigned the role of the purchaser with the ability to make payments to

facilities and CHMT for the PBF incentivised services based on their performances as indicated in the verified PBF invoices.	county health management team, county treasury, implementing partners, health facility management committees (FMCs) and civil society) was assigned the role of the purchaser although it was not actually making the payments for the PBF incentivised services, as this was done by the national fund holder (HSSF unit). Thus, its role was mainly overseeing county PBF implementation and, approving the PBF invoices and submitting them to the national fund holder (HSSF unit) for direct payments to the bank accounts of the PHC facilities and CHMT.	the PHC facilities depending on their verified performance in the quantity and quality indicators. This was because, in this policy design, the county treasury was to authorise the CDoH to open a ring-fenced county health special purpose account (SPA) where the PBF funds would be deposited and managed from jointly by the CDoH and county treasury.
4. Verifiers-responsible for verification of the accuracy of the self-reported performance indicators.	Internal verification- This role was assigned to a joint verification team (JVT) (comprising of a minimum of one local implementing partner and three members from the CHMT). Specifically, the JVT would: a) compare the PHC facilities PBF submitted invoices with service utilisation records in the facility registers and b) use a quality assessment tool to assess the quality of service delivery.<br><br>External verification- This role would be done by an independent (actor from outside the public health system) verifier contracted by the MoH and their role would be to conduct periodic sample based external verification using standard protocols provided by the MoH.<br><br>Community verification- This role would be done by a contracted community-based organisation and it would mainly involve comparing facility records with patient reports in the community.	Internal verification- This role was assigned to a joint verification team (JVT) (comprising of a minimum of one local implementing partner and three members from the CHMT). Specifically, the JVT would: a) compare the PBF submitted invoices with service utilisation records in the facility registers and b) use a quality assessment tool to assess the quality of service delivery.<br><br>External verification- This role would be done by an independent (actor from outside the public health system) verifier contracted by the MoH and their role would be to conduct periodic sample based external verification using standard protocols provided by the MoH.<br><br>Community verification- This role would be done by a contracted community-based organisation and it would mainly involve comparing facility records with patient reports in the community.
5. Providers- responsible for providing the incentivised services.	Public and faith-based PHC facilities that, among other things, could deliver at least six of the ten incentivised quantity indicators.	Public and faith-based PHC facilities that, among other things, could deliver at least six of the ten incentivised quantity indicators.
