# Supplementary material for: Political Prioritisation for Performance-Based Financing at the County Level in Kenya: 2015 to 2018
Source: Int J Health Policy Manag. 2023 Feb 14;12:6909. doi: 10.34172/ijhpm.2023.6909 (PMC10125155; doi:10.34172/ijhpm.2023.6909)
Supplement: Supplementary file 2 — Overview of Documents Included in the Case Study (n = 19). [file ijhpm-12-6909-s002.pdf]

Article title: Political Prioritisation for Performance-Based Financing at the County Level in Kenya: 2015 to 2018

Journal name: International Journal of Health Policy and Management (IJHPM)

Authors' information: Dennis Waithaka[1]*, Lucy Gilson[2,3], Edwine Barasa[1,4], Benjamin Tsofa[5,4], Marsha Orgill[2]

[1]Health Economics Research Unit, KEMRI-Wellcome Trust Research Programme, Nairobi, Kenya.

[2]Health Policy and Systems Division, School of Public Health and Family Medicine, University of Cape Town, Cape Town, South Africa.

[3]Department of Global Health and Development, Faculty of Public Health and Policy, London School of Hygiene and Tropical Medicine, London, UK.

[4]Centre for Tropical Medicine and Global Health, Nuffield Department of Medicine, University of Oxford, Oxford, UK.

[5]Health Systems Research Group, KEMRI-Wellcome Trust Research Programme, Kilifi, Kenya.

(*Corresponding author: DWaithaka@kemri-wellcome.org)

Supplementary file 2. Overview of Documents Included in the Case Study (n = 19).

Document description	Type of document
1. The Public Financial Management Act, 2012. Author: Government of Kenya [29]	Legislation
2. County Allocation of Revenue Act (CARA) of 2015. Author: National treasury [38]	Legislation
3. Financing agreement between International Development Agency and the republic of Kenya [39]	Financing agreement
4. PBF scale up 2014 – 2016, September 2014. Author: MoH [27]	Operational manual- early version
5. PBF scale up 2015-2018, April 2017. Author: MoH [40]	Operational manual- final version
6. National guidelines on the transfer of conditional grants (such as PBF) to county governments, August 2015. Author: National treasury [41]	National operational guidelines
7. Kilifi county financial guidelines on disbursement, use and reporting PBF, published in 2017. Author: MoH [42]	County operational guidelines
8. Capacity building for PBF scale up: invitation to facilitate as PBF master trainer of trainees (TOT), August 2015. Author: MoH [43]	Memo
9. Capacity building for PBF scale up: Nominees for PBF trainer of trainees (TOT) workshop 14th-18th September at the Kenya School of Government, Nairobi, August 2015. Author: MoH [44]	Memo
10. Capacity building for PBF scale up: Cascading PBF training in the counties, January 2016. Author: MoH [45]	Memo

11. PBF progress update for the financial year 2015/2016. August 2016. Author: MoH [46]	Memo
12. PBF procurement guidelines for essential equipment. January 2017. Author: MoH [47]	Memo
13. Kilifi sub-county health managers appointment letters to the Joint verification Team. June 2016 and January 2018. Author: Kilifi County Department of Health [48]	Letter
14. The piloting and scaling up Performance-Based Financing (PBF) in Healthcare in a Devolved Governance System: Experiences from Kenya between July 2011 and May 2015. Author: Alliance for Health Policy and Systems Research (AHPSR) [49]	Implementation report
15. Implementation completion and results report: The Kenya Health Sector Support Project (KHSSP), 2019. Author: World Bank [50]	KHSSP evaluation report
16. Technical assessment of the PBF pilot project in Samburu county, Kenya [51]	PBF pilot quantitative evaluation report
17. Evaluation of PBF pilot project in Samburu county, Kenya [52]	PBF pilot qualitative evaluation report
18. RBF Health Kenya,2014. Author: World Bank Group [53]	Website
19. Kenya receives 2.5 billion Kenyan Shillings to improve healthcare, October 2016. Author: The Standard- Health [54]	Website
