# Supplementary material for: Political Prioritisation for Performance-Based Financing at the County Level in Kenya: 2015 to 2018
Source: Int J Health Policy Manag. 2023 Feb 14;12:6909. doi: 10.34172/ijhpm.2023.6909 (PMC10125155; doi:10.34172/ijhpm.2023.6909)
Supplement: Supplementary file 3 — Interview Guide. [file ijhpm-12-6909-s003.pdf]

Article title: Political Prioritisation for Performance-Based Financing at the County Level in Kenya: 2015 to 2018

Journal name: International Journal of Health Policy and Management (IJHPM)

Authors' information: Dennis Waithaka[1]*, Lucy Gilson[2,3], Edwine Barasa[1,4], Benjamin Tsofa[5,4], Marsha Orgill[2]

[1]Health Economics Research Unit, KEMRI-Wellcome Trust Research Programme, Nairobi, Kenya.

[2]Health Policy and Systems Division, School of Public Health and Family Medicine, University of Cape Town, Cape Town, South Africa.

[3]Department of Global Health and Development, Faculty of Public Health and Policy, London School of Hygiene and Tropical Medicine, London, UK.

[4]Centre for Tropical Medicine and Global Health, Nuffield Department of Medicine, University of Oxford, Oxford, UK.

[5]Health Systems Research Group, KEMRI-Wellcome Trust Research Programme, Kilifi, Kenya.

(*Corresponding author: DWaithaka@kemri-wellcome.org)

Supplementary file 3. Interview Guide.

Supplementary file 3. Interview Guide

**Date of discussion:**	**Interviewer:**
**Venue:**	**Note taker:**
**Time start:**	**Interviewee's Code:**
**Time stop:**	
**Interview completed (Yes/No)**	
**Reason for incomplete interview**	

Interviewer's remarks about the session and issues from debrief

1.

2.

3.

The interview will be conducted online after the participant has been taken through the consent form, signed it and has verbally agreed to participate. The interview will take place in a private location chosen by the interviewee. Before starting the interview, the student researcher will respond to any questions that the interviewee may have. See the guiding questions and prompts in the next page:

Guiding questions

- How and when did you hear that PBF would be introduced into the county?
  - Probe for key actors. For example, if heard in a workshop or meeting, probe for the actors who were there and the roles they played
  - Probe for key events. For example, what was happening in the county at the time?
- From whom did you hear the news about PBF and what information were you given? [speaks to ideas and issue characteristics]
  - For example, what information were you given on why PBF was being implemented in Kilifi?
- What was your role at the time in the county?
  - At what level of the health system? Global, national or local
  - Probe for source of power. In terms of their position, resources under their control, expertise and networks (who they work closely with)
- At the time what difference did you believe PBF would make in the county? Please explain [speaks to ideas]
  - Probe for the source of information or evidence on the beliefs
- Was this belief / disbelief shared amongst your colleagues? If yes or no, can you explain why? [speaks to shared ideas]
- Have these beliefs changed over time? please explain why you think so [examining for evolution of ideas]
  - Probe for the source of information or evidence on the change in beliefs

- Did you play any role in the introduction or implementation of PBF in the county? Please explain your role(s)
- Which actors would you say had the most influence in the introduction of PBF in Kilifi County? Why those actors? [speaks to actor power]
    - Probe for source of power. Is it due to their positions, resources, knowledge or networks/relationships?
- Which actors would you say had surprisingly low influence in the introduction of PBF in Kilifi County? Why those actors? [speaks to actor power]
    - Probe for source of power. Is it due to their positions, resources, expertise or networks/relationships?
- Did the influence of the identified actors over PBF change over time? If yes, how and why? [speaks to changes in actor power]
- Were there actors who did not want PBF or certain aspects of PBF? If yes, please explain why you say so? [speaks to any contestations]
    - Did their views towards PBF change over time? Why do you say so?
- Could you please explain where the PBF funds come from and the process through which they reach the facilities?
    - Does the national and county government contribute to the funding of PBF? If yes or no, why do you think that is the case? [speaks to outcome of political priority]
    - Who is/was meant to manage the funding for PBF?
    - Was there any specific reactions from health managers, health providers and/or politicians to the proposed funding arrangements?
    - Were there any contestations around funding?
- Apart from funds, are there any resources (human and technical) that the national and Kilifi County government allocates towards PBF? If yes, which and when? If not, why do you think they do not allocate any resources? [speaks to outcome of political priority]
- Are there any local policy documents or laws related to PBF that have been developed in Kilifi County? [speaks to outcome of political priority]
    - If yes, which ones and when were they developed? Who were involved in developing them? What processes were involved in making them?
    - If no, why do you think there are no local policy documents or laws?
- Where is the county now in relation to implementing PBF? What is currently happening?

Finally, is there anyone you would suggest that I speak to as well? [snowballing]

Thank you for your time. If it is fine with you, I will be in touch if there is need to follow-up issues for clarification. I will also share my findings with you at a later stage.
